# Effect of a Pleuran-Based Supplement on Salivary IgA Secretion in Children With Recurrent Respiratory Infections

**DOI:** 10.1016/j.curtheres.2025.100780

**Published:** 2025-02-15

**Authors:** Peter Kunc, Jaroslav Fabry, Michaela Matiscakova, Katarina Istvankova, Zuzana Diamant, Juraj Majtan, Milos Jesenak

**Affiliations:** 1 Clinic of Paediatric Respiratory Diseases and Tuberculosis, National Institute of Paediatric Tuberculosis and Respiratory Diseases, Dolny Smokovec, Jessenius Faculty of Medicine, Comenius University in Bratislava, Bratislava, Slovakia; 2 Department of Pathological Physiology, Jessenius Faculty of Medicine, Comenius University in Bratislava, University Teaching Hospital, Martin, Slovakia; 3 Department of Clinical Pharmacy and Pharmacology, University of Groningen, Groningen, The Netherlands; 4 Department of Respiratory Medicine, First Faculty of Medicine, Charles University and Thomayer Hospital, Prague, Czech Republic; 5 Department of Microbiology, Immunology and Transplantation, KU Leuven, Catholic University of Leuven, Leuven, Belgium; 6 Department of Microbial Genetics, Institute of Molecular Biology, Slovak Academy of Sciences, Bratislava, Slovakia; 7 Department of Microbiology, Faculty of Medicine, Slovak Medical University, Bratislava, Slovakia; 8 Department of Pediatrics and Adolescent Medicine, Jessenius Faculty of Medicine, Comenius University in Bratislava, University Teaching Hospital in Martin, Martin, Slovakia; 9 Institute of Clinical Immunology and Medical Genetics, Jessenius Faculty of Medicine in Martin, Comenius University in Bratislava, University Hospital Martin, Martin, Slovakia

**Keywords:** Allergy, Children, Mucosal immunity, Respiratory tract infections, Salivary sIgA, ß-glucan

## Abstract

**Background:**

ß-glucans isolated from natural sources have demonstrated pluripotent immunomodulatory potential, making them a promising supportive treatment for the management of recurrent respiratory infections (RRIs) in children. This study aimed to evaluate the effects of a pleuran-based supplement (ß-glucan isolated from *Pleurotus ostreatus* in combination with vitamin D and zinc) on mucosal immunity –through modulating salivary secretory immunoglobulin A (sIgA) levels –in children with RRIs.

**Methods:**

This monocentric, prospective, open-label pilot study investigated the effect of an orally administered pleuran/vitamin D/zinc supplement (1–2 chewable tablets daily depending on body weight) on the dynamics of sIgA secretion measured in saliva samples collected at three timepoints: at baseline and after 4–6 and 8–10 days.

**Results:**

This study included 49 children aged 6-11 years (mean age: 8.2 ± 1.6 years) with a history of one or more of the following conditions in the inclusion criteria: RRIs, allergy, and asthma. After 8–10 days with daily administration of the chewable pleuran/vitamin D/zinc supplement, children exhibited a statistically significant increase in salivary sIgA concentrations compared with baseline (227 ± 211 µg/mL; *P* = 0.045). No adverse events were observed during the course of the study in relation to the administration of pleuran-based supplement.

**Conclusions:**

We demonstrated the beneficial effects of the short-term administration of a pleuran-based chewable supplement on mucosal immunity through increasing salivatory sIgA levels. This study confirms the favourable safety profile of this pleuran/vitamin D/zinc combination, which could be beneficial for children with acute or recurrent respiratory infections, including children with allergies and/or asthma. Moreover, the significant increases in salivary sIgA concentrations that were observed after a few days of supplementation support the use of pleuran in not only the prevention but also the treatment of acute respiratory infections.

## Graphical Abstract

**Figure:**
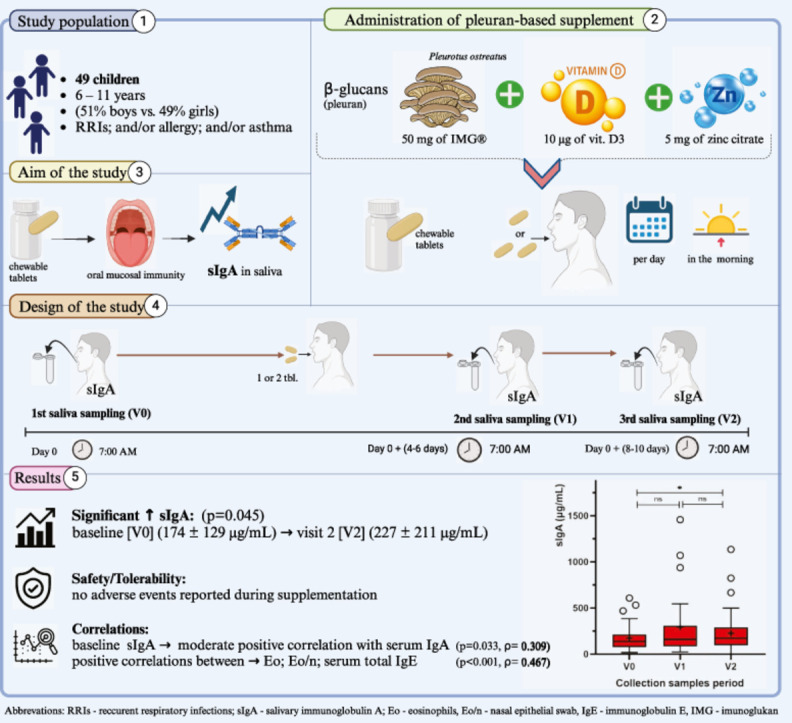

## Introduction

Recurrent respiratory infections (RRIs) impose high medical and socioeconomic burdens on society. RRIs affect the upper and lower respiratory tract, and approximately 25% of children under 1 year of age and 6% of children during their first 6 years of life experience RRIs.^1^ However, studies show that the frequency of RRIs declines in children with increasing age. A combination of intrinsic and extrinsic factors contributes to the increased susceptibility to respiratory infections that is observed in preschool-aged children. In particular, immature mucosal immunity is a key contributing factor to this susceptibility.^2^

RRIs are predominantly caused by viruses (85–95%) and are usually self-limiting. The key objective of a proper diagnostic approach to RRIs is the exclusion of primary immune defects, local anatomical factors, developmental anomalies, and focal infections, and also the exclusion of genetic disorders such as primary ciliary dyskinesia or cystic fibrosis in the subgroup of children with more severe and persisting symptoms.^3^ Uncomplicated RRIs are among the leading causes of frequent outpatient visits to primary care providers, and they result in unnecessary medical examinations. This overuse of healthcare resources imposes a significant burden on an already strained healthcare system. Frequent healthcare visits also increase antibiotic prescribing in children.^4^

Allergic background contributes significantly to the increased rate of respiratory tract infections in children and can modulate the mucosal immune response and functions. Currently, new emerging tools (e.g., computational immunoinformatic studies) are arising in detecting the novel potential allergens or contributing factors to the general burden of respiratory allergic diseases and can be applied for the development of novel therapeutic strategies (e.g., immunomodulators, peptide vaccines).^5^

Immunoglobulin A (IgA) plays an essential role in maintaining the proper functioning and homeostasis of mucosal immunity. IgA is critical for maintaining the primary barrier function of the gastrointestinal and respiratory mucosal surfaces, and it acts as an important first line of defense against the invasion of pathogens.^6^ Produced by plasma cells after antigenic stimulation, IgA is the predominant antibody isotype present at respiratory tract mucosal surfaces. It exists in two subclasses, namely IgA_1_ and IgA_2_, which can be produced in both monomeric and dimeric forms. The predominant form, secretory IgA (sIgA), is a dimer that is most abundantly found in mucosal secretions such as saliva, tears, sweat, and gastrointestinal fluids. sIgA also participates in maintaining microbial homeostasis and diversity in the intestinal mucosa.^7^

Biologically active polysaccharides such as ß-glucans (i.e.*,* glucose polymers prevalent in fungi, yeasts, bacteria, and cereals) are amongst the most studied of the compounds that are natural immunomodulators with pleiotropic biological effects.^8^ The immunomodulatory potential of ß-glucans varies depending on their sources, degrees of purification, structural characteristics (including degree of branching), solubility, and molecular conformations. The immunomodulatory effects of ß-glucans are mediated by several different receptors that are expressed on the surface of both immune and non-immune cells.^9^ Dectin-1 –which is predominantly expressed on innate immune cells such as macrophages, neutrophils, and dendritic cells –is considered the most important receptor in mediating the biological activities of β-glucans.^10^ β-Glucans synergistically activate toll-like receptors (TLR2 and TLR4) and Dectin-1 in human mucosal dendritic cells and other innate immune cells that recognize pathogen-associated molecular patterns (PAMPs).^11^^,^^12^ This combined activation leads to dynamic changes in intracellular signaling pathways and the production of specific cytokines. These mechanisms represent one of the main methods of acquiring immunological memory in the non-specific part of immune system, which is known as trained immunity.^13^ The long-term epigenetic and metabolic reprogramming of cells can contribute to more potent immune responses.^14^ It has been proposed that insoluble ß-glucans directly interact with immune cells within the Peyer's patches in the small intestine after immune cell activation.^15^ Many studies have revealed various activities of β-glucans such as increasing NK cell numbers, stimulating phagocytic activity, enhancing antibody development, supporting T-cell activity, and improving the post-vaccination response.^16^ According to the pluripotent biological effects of β-glucans, these naturally-derived compounds can be useful in treating children experiencing RRIs. The β-glucan that has been clinically studied the most in children with RRIs is insoluble beta- (1,3/1,6)-d-glucan - pleuran; this molecule is isolated from the edible mushroom *Pleurotus ostreatus* and has been reported to decrease respiratory morbidity rates.^17^, ^18^, ^19^ Moreover, its anti-infective and anti-allergic effects could protect children with RRIs and partly controlled perennial asthma against the worsening of allergic and/or asthmatic symptoms.^20^^,^^21^

There is a scarcity of knowledge about the relationship between the oral administration of β-glucans and the production of sIgA. Therefore, in this monocentric, prospective, open-label pilot study we investigated the potential of a pleuran-based supplement, including vitamin D and zinc and administered in a novel chewable tablet, to induce the synthesis and secretion of sIgA in children.

## Materials and Methods

### Study population

This monocentric, prospective, open-label pilot study was conducted at the National Institute of Paediatric Tuberculosis and Respiratory Diseases (Dolny Smokovec, Slovakia). The study population consisted of children aged 6–11 years who were admitted to the pulmonary department for the management of chronic respiratory diseases. Eligible individuals with RRIs needed to meet at least one of the criteria of De Martino for inclusion^2^: experiencing six or more respiratory infections per year, having at least one upper respiratory tract infection per month from September to April, or experiencing at least three lower respiratory tract infections annually. Other inclusion criteria included having a diagnosis of allergic rhinitis or having mild to moderately severe allergic asthma that was well controlled. We excluded patients with any of the following conditions: an ongoing respiratory infection, autoimmune disease, an active malignancy, significant malnutrition, primary immunodeficiency (particularly selective IgA deficiency), severe secondary immunodeficiencies, uncontrolled asthma, or oral trauma (e.g., ulcerations, aphthae, or recent tooth extraction). Children who were currently receiving or had received any antibiotics or immune-interventional therapies (immunomodulatory agents and specific allergen-immunotherapy) within 14 days before enrolment were excluded from the study. This study included only children whose parents or legal guardians provided written informed consent. The research protocol was approved by the Ethics Committee of the National Institute of Paediatric Tuberculosis and Respiratory Diseases (ID0102303). All research procedures involving human participants adhered to the ethical principles established in the Declaration of Helsinki (revised version 2000.09.01) and were performed in full accordance with the rules for clinical testing in the Slovak Republic.

### Study design

The primary endpoint of this open-label pilot clinical trial was to evaluate the effects of a pleuran-based supplement (Imunoglukan P4H® chewable tablets) on the induction of sIgA secretion during an 8–10-day oral-administration period. Each tablet contained 50 mg of the active substance IMG®, 5 mg of zinc (zinc citrate) and 10 µg of vitamin D3 (cholecalciferol). IMG®, which is the active ingredient in this natural product, is a complex of biologically active polysaccharides consisting of ß-1,3/1,6-d-glucan pleuran that was isolated from *Pleurotus ostreatus* using patented technology; this active substance was previously identified and thoroughly characterised by Karacsonyi and Kuniak.^22^ The dose of product that was administered depended on the weight of each participant: children weighing less than 25 kg took one tablet daily at the same time in the morning for up to 10 days, whereas children weighing 25 kg or more took two of these tablets (100 mg of IMG® + 10 mg of zinc + 20 µg vitamin D) once daily for up to 10 days. The investigated product was administered to participants in a novel galenic formulation of chewable tablets to facilitate the direct interaction of the product's active ingredients with the lymphatic tissue within the oral mucosa.

**Fig. 1:**
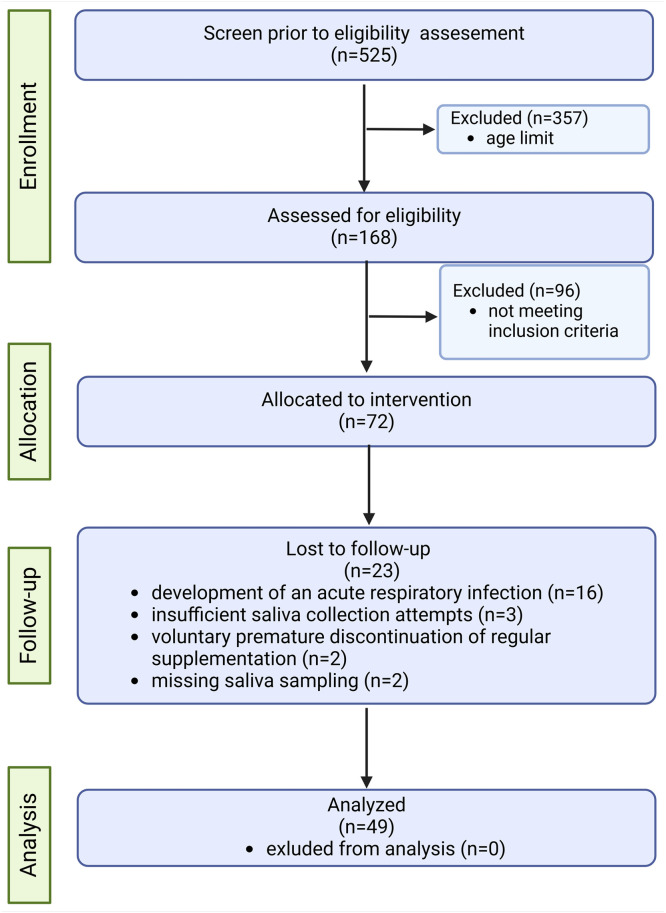
CONSORT flow diagram of the selection process of the study participants (created in Biorender.com).

The secondary endpoint of the study was to assess the safety and tolerability of the administered product; if an adverse event occurred, the attending physician was required to fill out an “Adverse event form.” In addition, selected immunological parameters were analyzed on day 0 (V0): serum immunoglobulins of classes G, A, M, and E (IgG, IgA, IgM, and IgE); eosinophils in peripheral blood (Eo), and a nasal epithelial swab (Eo/n). Fig. 1 illustrates the study flow, detailing participant recruitment, reasons for drop-out, administration of study intervention, and sample collection procedures.

### Sample size calculation

We calculated the number of study patients according to sIgA mean value. In a previous study,^23^ the mean and standard deviation (SD) of the sIgA values in children with recurrent respiratory infection were 49 µg/mL and 25 µg/mL, respectively. We assumed that the sIgA mean of the group of patients, where chewable pleuran/vitamin D/zinc supplement was administered, is 20% higher than the mean value of sIgA in published study. With 80% power and a type I error of 5%, the estimated sample size was 41.

## Methods

Study participants were instructed by investigators to collect their first saliva sample for the detection of sIgA before taking the pleuran-based supplement in the morning, on an empty stomach, and by following the instructions of healthcare personnel: collection was to be performed in morning before performing oral hygiene, after rinsing the mouth with water, and while in a state of physical and psychological rest. Saliva was collected into prelabelled tubes (PYREX® disposable glass conical centrifuge tubes, 10 ml). Saliva samples (minimum volume of 2 mL) were centrifuged at 3,000 rpm for 15 min and subsequently stored at –20°C. The Sandwich ELISA method (IgA Saliva ELISA Kit, DiaMetra S.r.l, Italy) was used to determine sIgA concentrations.

After the collection of the first baseline sample (V0), participants received the initial dose of chewable pleuran/vitamin D/zinc combination tablets. Participants adhered to a consistent regimen, consuming the prescribed doses of tablets at the same time each morning throughout the study period. Following the procedure used for V0, a second saliva sample was collected between days 4 and 6 before the morning dose of the administered product (V1) and a final third saliva sample was collected between days 8 and 10 after the beginning of the treatment (V2). The health status of each participant was repeatedly monitored throughout the study, as was their adherence to treatment. All participants were routinely evaluated by medical staff, and any children who showed signs of an early respiratory infection during the study period were excluded from the study.

### Statistical analyses

Continuous variables are presented as mean and standard deviation (SD). Based on the results of a normality test, the Wilcoxon test was used to compare continuous values between the baseline examination (V0) and the other two examinations (V1 and V2). Correlation analyses were performed using Spearman's correlation. Statistical significance in all statistical tests was defined as *P* < 0.05. Data were analyzed using IBM SPSS Statistics for Windows version 20 software (IBM Corp, Armonk, NY, USA).

## Results

### Primary endpoints –sIgA secretion

This pilot open-label study enrolled 72 children out of 525 children who were admitted to the study pulmonary department due to RRIs and met the inclusion criteria for the study enrolment. A total of 49 children aged 6–11 years (mean age: 8.2 ± 1.6 years) were included in the final data analysis. 23 children were lost to follow-up prematurely due to a development of an acute respiratory infection (*n* = 16), insufficient saliva collection attempts (*n* = 3), voluntary premature discontinuation of regular supplementation (*n* = 2) or missing saliva sampling (*n* = 2). There was an almost even gender distribution (51% boys vs. 49% girls). Exposure to tobacco smoke in households was reported for nearly 31% of the participants. Most participants (69%) had one sibling; approximately 12% of households were one-child families, and three-child families represented a small minority (less than 2%). A high proportion (85.7%) of participants reported having allergic conditions, with allergic rhinitis being the most frequent (85.7%), followed by bronchial asthma (46.9%) and atopic eczema (2%). Antihistamines were the most commonly used antiallergic drugs (83.7%), followed by intranasal corticosteroids. The short-acting β₂-agonist salbutamol was the anti-asthmatic medication most commonly used by participants with asthma (34.7%), followed by inhaled corticosteroids (28.6%). Table 1 summarizes the key baseline demographic characteristics and selected clinical features of the whole study population and a sub-population that was referred to as super-responders. A super-responder was defined as a participant with sIgA values higher than the 95% confidence interval and with a difference between V0 and V1 value of sIgA that was higher than 50%.

**Table 1:** Baseline demographic characteristics of the whole study population (N = 49) and the subpopulation defined as super-responders (N = 7).

	Parameter	Whole study population (N)	Whole study population (%)	Super-responder sub-population (N)	Super-responder sub-population (%)
Gender					
	Male	25	51	4	57.1
	Female	24	49	3	42.9
Age (years)		8.2 ± 1.6	-	9.3 ± 1.5	-
Passive smoking		15	30.6	2	28.6
Number of siblings		1.1 ± 0.6	-	1 ± 0.6	-
Allergy					
	Allergic rhinitis	42	85.7	6	85.7
	Atopic eczema	1	2.0	0	0
	Bronchial asthma	23	46.9	3	42.9
Treatment					
	AH	41	83.7	6	85.7
	INCS	32	65.3	5	71.4
	Topical AH (nose and eyes)	9	18.4	0	0
	ICS	14	28.6	1	14.3
	ICS/LABA	8	16.3	2	28.6
	SABA	17	34.7	3	42.9
	LTRA	2	4.1	0	0

AH = antihistamines; INCS = intranasal corticosteroids; ICS = inhaled corticosteroids; LTRA = leukotriene receptor antagonists; LABA = long-acting beta-agonists; SABA = short-acting beta-agonists.

**Fig. 2:**
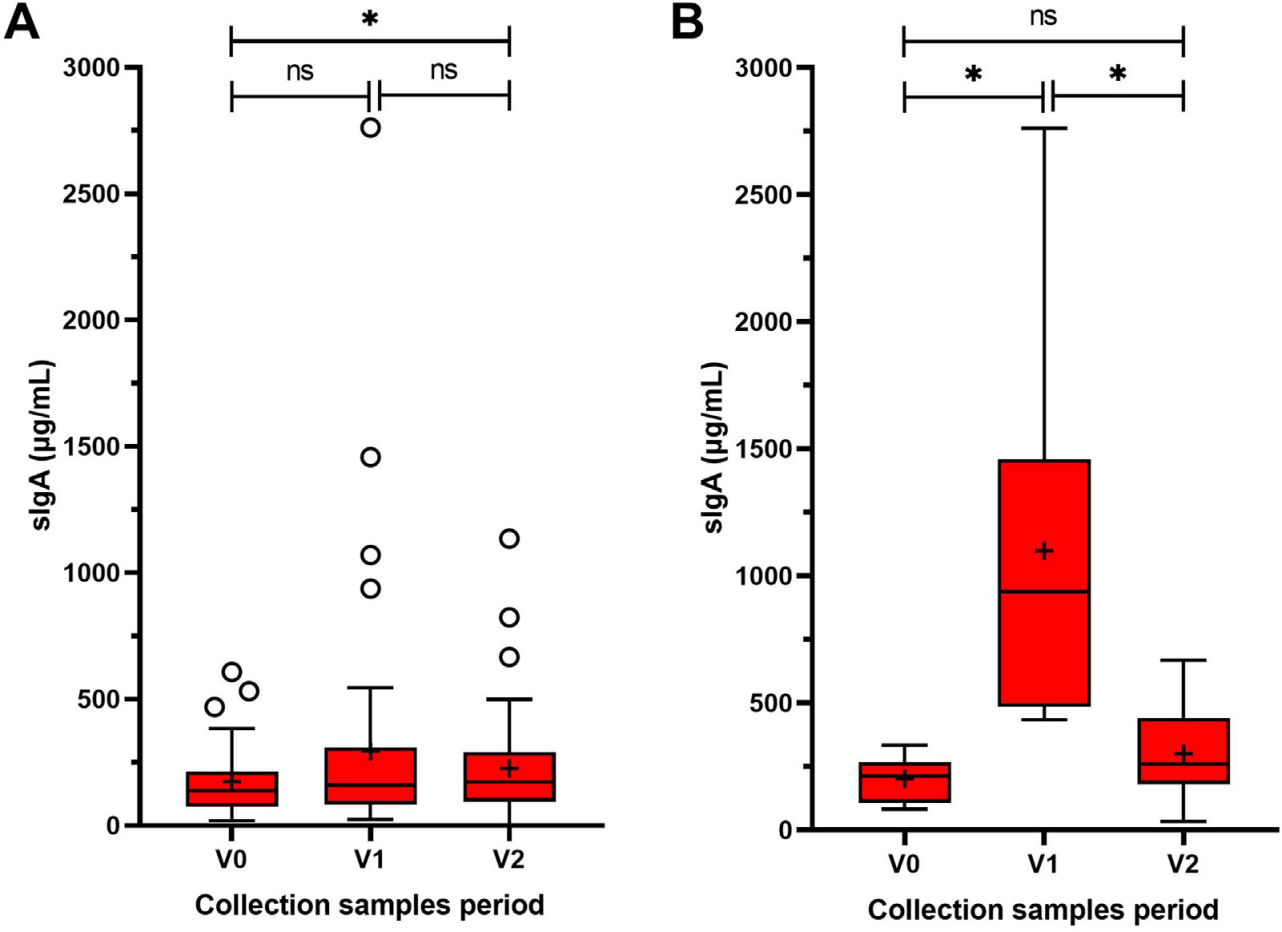
Tukey box plot of secretory immunoglobin A (sIgA) concentration values measured at V0 (baseline), V1, and V2 in (A) all participants and (B) seven participants who exhibited a rapid and high response to the intervention and were referred to as “super-responders.” The box indicates the lower and upper quartiles, the whiskers represent the 10^th^ and 90^th^ percentiles, and the horizontal line in each box is the median. Open circles indicate “outlier” values. The Wilcoxon test was used to compare measured sIgA concentrations between the three visits. *P < 0.05; ns, nonsignificant.

The concentration of sIgA in saliva from participants was measured at three time points. The first saliva sample (V0) showed a mean sIgA concentration of 174 ± 129 µg/mL. The second sample (V1) showed a statistically insignificant increase compared with V0, with a mean sIgA concentration of 295 ± 455 µg/mL (*P* = 0.242). However, the final sample (V2) revealed a statistically significant increase in sIgA concentration compared with baseline, with a mean sIgA concentration of 227 ± 211 µg/mL (*P* = 0.045) Descriptive analysis identified seven participants who exhibited a rapid and high response to the intervention and were referred to as “super-responders.” Among the super-responders, the highest sIgA concentrations were observed in V1 (2762 µg/ml) and V2 (668 µg/ml), as shown in Fig. 2.

### Secondary endpoints –tolerability and safety, analysis of selected laboratory parameters

Administration of the pleuran/vitamin D/zinc supplement was well-tolerated and did not result in any adverse events throughout the study period. This confirms a favorable safety profile for this immunomodulatory intervention.

Evaluation of the relationships between the sIgA concentration at V0 and various laboratory parameters (serum IgG, IgA, IgM, total IgE, Eo and Eo/n) showed the following findings: sIgA in saliva collected at V0 displayed a statistically significant moderate positive correlation with serum IgA (Spearman's rho = 0.309, *P* = 0.033). Analysis of non-specific atopy markers demonstrated some positive associations. A moderate positive correlation was found between Eo/n and serum IgE (Spearman's rho = 0.514, *P* < 0.001). Additionally, significant positive correlations were found between Eo and Eo/n (Spearman's rho = 0.467, *P* < 0.001) and between Eo and serum total IgE (Spearman's rho = 0.483, *P* < 0.001). These findings are summarized in Table 2. Analysis of serum immunoglobulin levels in participants did not show concentrations below the reference range for any of the evaluated classes (IgG, IgA, and IgM).

**Table 2:** Correlation coefficient (r) and statistical significance (parenthesis) values calculated by Spearman's correlation analysis.

	ser_IgA	ser_IgG	ser_IgM	ser_IgE	Eosinophils
ser_IgE	0.196 (0.181)	0.114 (0.444)	0.028 (0.851)	1	0.483⁎⁎ (0.001)
Eosinophils	0.085 (0.568)	0.059 (0.696)	0.212 (0.153)	0.483⁎⁎ (0.001)	1
Eo/n	0.125 (0.398)	0.137 (0.360)	0.005 (0.975)	0.514⁎⁎ (0.000)	0.467⁎⁎ (0.001)
sIgA_V0	0.309* (0.033)	0.188 (0.206)	0.170 (0.253)	−0.097 (0.511)	−0.057 (0.698)

ser_IgG = serum immunoglobulin G; ser_IgA = serum immunoglobulin A; ser_IgM = serum immunoglobulin M; ser_IgE = serum immunoglobulins class E; sIgA = secretory immunoglobulin A; Eo = eosinophils in peripheral blood; Eo/n = eosinophils in nasal epithelial swab.

⁎ Correlation is statistically significant at the 0.05 level.

⁎⁎ correlation is statistically significant at the 0.001 level.

## Discussion

RRIs are a frequent cause of childhood healthcare visits that often lead to overdiagnosis and unnecessary medical interventions (antibiotics). Increased morbidity in young children, particularly before school age, is believed to be a consequence of the dynamic maturation and training of the immune system as it encounters various pathogens. The mucosal immune system, which forms a crucial protective barrier against external threats, plays a central role in the host's defense against airborne pathogens. sIgA is a major humoral component produced by mucosal plasmocytes that is a key player in this defence.^2^, ^3^, ^4^^,^^6^

The pluripotent immunomodulatory properties of ß-glucans from various sources are generally well-established. The immunomodulatory effects of ß-glucans are influenced by their structural variations, including the type of glycosidic linkages and branching patterns they contain.^10^ ß-glucans show promising effects as potential vaccine immune-adjuvants in addition to lowering cholesterol, regulating blood sugar levels, and protecting against the negative effects of environmental toxicity.^24^ Several studies support the clinical efficacy of insoluble beta- (1,3/1,6)-d-glucan pleuran isolated from *Pleurotus ost*reatus in reducing the frequency of respiratory infections. These studies demonstrate that pleuran can decrease the incidence of specific respiratory infection subtypes, can potentially mitigate symptom intensity, and can accelerate recovery time.^17^, ^18^, ^19^ Pleuran also showed potential as an anti-allergic agent in clinical studies that demonstrated a substantial reduction in peripheral blood eosinophilia, a stabilization of total serum IgE levels, and the relief of allergic symptoms after oral administration.^20^ Pleuran used as an add-on therapy to GINA-based standard-care treatment showed efficacy in improving asthma control and preventing asthma exacerbations and RRIs in children with partially controlled perennial asthma.^21^ Furthermore, all previous studies reported a favorable safety and tolerability profile of pleuran in children with RRIs, allergy, or bronchial asthma.^17^, ^18^, ^19^, ^20^, ^21^ Zinc is a vital trace element that plays an essential role in immune function and homeostasis. It acts as a critical regulator in the maturation and differentiation of immune cells, modulates inflammatory responses, and supports the integrity and functional capacity of epithelial barriers.^25^ Similarly, vitamin D –another essential micronutrient and immunomodulator –plays a pivotal role in maintaining homeostasis. In the context of the mucus layer and the underlying epithelium, the underlying epithelium safeguards an appropriate level of antimicrobial peptides in the mucus and supports epithelial integrity by strengthening intercellular junctions. It also enhances immune cell proliferation and modulates immune responses by suppressing Th1/Th17 cells and activating Treg cells.^26^

Although several human studies have investigated the link between ß-glucan administration and increased sIgA production, the findings remain inconsistent. The administration of yeast-derived ß-glucans to children and older adults did not produce statistically significant changes in the secretion of sIgA in previous studies.^27^^,^^28^ On the contrary, other controlled clinical trials had findings similar to ours and reported an increase in sIgA production after the administration of ß-glucans derived from different sources under resting conditions or after intense exercise.^29^^,^^30^ This heterogeneity in results likely arises from significant variations in study design. Key factors contributing to this variability include differences in participant characteristics (age, gender, and comorbidities), ß-glucan sources, processing methods and purity of the final products, and actual doses. In particular, Lehne et al.^29^ demonstrated a dose-dependent effect of ß-glucan on sIgA formation in healthy adults, highlighting the importance of this factor. Some positive effects of vitamin D supplementation on salivary sIgA secretion have been observed in patients with vitamin D deficiency or in patients under physical training conditions in which stress levels are increased.^31^^,^^32^ The use of a zinc-fortified formula in infants during rehabilitation can prevent the development of zinc deficiency and improve growth and immune function by increasing sIgA concentrations.^33^

There is little knowledge about the effects of the oral administration of the β-glucan pleuran in combination with vitamin D and zinc on the production of sIgA. The extraction and purification processes used for ß-glucans are recognized as critical determinants of their immunomodulatory efficacy. This variability in processing methods probably contributes to the inconsistencies observed in research findings related to the described ß-glucan effects. In this monocentric, prospective, open-label pilot study we hypothesized that this intervention would enhance mucosal immunity by promoting sIgA production in compromised children. After 8–10 days of immunomodulatory supplementation, salivatory sIgA concentrations exhibited a statistically significant increase compared with baseline levels (*P* = 0.045). Most of the study participants had been diagnosed with allergic diseases, such as allergic rhinitis or allergic bronchial asthma. Moreover, the significant increase of sIgA within a few days of supplementation supports the use of pleuran in both the prevention and treatment of acute respiratory infections. Additional analyses in this study investigated the relationships between selected laboratory parameters. We observed statistically significant positive associations between baseline salivary sIgA levels and the following measures: serum IgA levels, peripheral eosinophil counts, mucosal eosinophil counts, and serum total IgE levels.

In conclusion, this study pioneered the investigation of a supplement based on the ß-glucan pleuran (from *Pleurotus ostreatus*) on oral mucosal immunity in children with RRIs, including allergic and asthmatic children. However, study limitations, including the relatively small sample size and the absence of a placebo-controlled group, mean that further research is required to confirm our findings and definitively attribute the changes we observed to the supplementation with ß-glucan in combination with vitamin D and zinc. Future directions include enrolling a larger and more diverse sample population (e.g., athletes and obese children), implementing a placebo-controlled design or a pleuran-based intervention without vitamin D and zinc, investigating the dose-dependent relationship between ß-glucan intake and sIgA production, and analyzing additional salivary immune parameters (such as pro-inflammatory cytokines and cortisol) to give a more comprehensive picture of the immune response. By addressing these aspects, future studies can better determine the potential of the *Pleurotus ostreatus*-derived ß-glucan pleuran to improve oral mucosal immunity, in addition to identifying other potential clinical applications.

## Data availability

Data will be made available on request.

## CRediT authorship contribution statement

**Peter Kunc:** Conceptualization, Data curation, Formal analysis, Investigation, Methodology, Project administration, Supervision, Writing – original draft, Writing – review & editing. **Jaroslav Fabry:** Methodology, Project administration, Writing – review & editing. **Michaela Matiscakova:** Methodology, Project administration, Writing – review & editing. **Katarina Istvankova:** Methodology, Project administration, Writing – review & editing. **Zuzana Diamant:** Conceptualization, Writing – review & editing. **Juraj Majtan:** Data curation, Writing – original draft, Writing – review & editing. **Milos Jesenak:** Conceptualization, Writing – original draft, Writing – review & editing.

## Declaration of competing interest

The authors declare that they have no known competing financial interests or personal relationships that could have appeared to influence the work reported in this paper.
